# Protective Effect of Rutin Trihydrate Against Dose-Dependent, Cisplatin-Induced Cardiac Toxicity in Isolated Perfused Rat’s Heart

**DOI:** 10.7759/cureus.21572

**Published:** 2022-01-24

**Authors:** Ishfaq A Bukhari, Osama Y Mohamed, Abdulrahman M Alhowikan, Rahmathunnisa Lateef, Hanan Hagar, Raghad A Assiri, Wa’ad Massoud A Alqahtani

**Affiliations:** 1 Department of Physiology, College of Medicine, King Saud University, Riyadh, SAU; 2 College of Medicine, Imam Mohammad Ibn Saud Islamic University, Riyadh, SAU

**Keywords:** oxidative stress, histology, cardiac function, rutin trihydrate, cisplatin

## Abstract

Background

Cisplatin is a common anticancer drug with potential cardiac and renal toxicities. Rutin, a natural compound present in various medicinal plants, has been shown to protect against chemotherapy-induced toxicities. In this study, we explored the protective effect of rutin against the dose-dependent cardiotoxic effects of cisplatin such as perfusion pressure, histopathologic effect on the myocardium, and oxidative stress in isolated perfused rat hearts.

Methodology

The cardiotoxic effects of cisplatin were studied at three dosages (1, 7, and 14 mg/L) in isolated perfused rat hearts. The dose-dependent, cisplatin-induced toxic effects on left ventricular pressure (LVP), heart rate (HR), dp/dt (maximum), dp/dt (minimum), perfusion pressure, pressure-time index, contractility index, and duration of diastole were assessed. The effects of cisplatin were measured one minute before perfusion of cisplatin and 60 minutes after perfusion of the isolated rat hearts.

Results

Cisplatin (1-14 mg/L) caused a significant (p < 0.05) dose-dependent reduction in LVP. The percentage LVP values reduced from 94 ± 9 (control untreated hearts) to 70 ± 6, 69 ± 5, and 65 ± 4 in hearts treated with 1, 7, and 14 mg/L of cisplatin, respectively. Similarly, cisplatin at similar doses caused a marked reduction in the values of dp/dt (maximum), dp/dt (minimum), and pressure-time index in isolated rat hearts. The respective percentage values of these parameters compared to those of untreated hearts were significantly reduced from 101 ± 7 to 72 ± 5, 92 ± 8 to 69 ± 4, and 92 ± 12 to 57 ± 7 in hearts treated with 14 mg/L of cisplatin. Perfusion of hearts with rutin trihydrate (1 µM/L) 10 minutes before administration of cisplatin and throughout the experiment attenuated the detrimental effects of cisplatin on cardiac functions in isolated rat hearts (p < 0.05). In addition, cisplatin-induced degeneration and necrosis of cardiac muscle cells reduced with the concurrent administration of rutin and restored normal heart histology. Moreover, cisplatin-induced reduction in glutathione and increased level of malondialdehyde in the myocardium was reversed by concurrent administration of rutin in isolated rat hearts.

Conclusions

Cisplatin produced a dose-dependent impairment of several parameters of cardiac function such as LVP, contractility index, and pressure-time index. It caused histopathological alterations in isolated rat hearts. These harmful effects of cisplatin were suppressed by rutin trihydrate, suggesting the potential protective effects of rutin against cisplatin-induced cardiotoxicity. Rutin trihydrate also improved the reduced glutathione contents and suppressed the malondialdehyde contents in the cardiac tissue of isolated rat hearts, suggesting that the observed beneficial effects of rutin trihydrate in this study could be related to its antioxidant properties.

## Introduction

Cisplatin is a broad-spectrum, anti-malignant drug widely employed in the management of testicular, ovarian, gastric, esophageal, and non-small-cell lung cancers, as well as hematological malignancies [1]. The clinical use of cisplatin is associated with severe toxicities affecting the cardiac, gastrointestinal, renal, neurological, and hematological systems [2]. Previous studies have reported multiple cardiotoxic effects of cisplatin such as unstable angina pectoris [3], bradycardia [4], acute myocardial infarction [5], atrioventricular block [6], and cardiomyopathy [7]. An increase in oxidative stress and a decrease in antioxidant enzymes are implicated in the cardiotoxic effects of cisplatin [8].

Rutin trihydrate, a bioflavonoid abundant in plants, is known for its beneficial effects against various drug-induced toxicities, including doxorubicin and cisplatin-induced cardiotoxicity and memory deficits [9,10]. The protective effects of rutin are attributed to its antioxidant and anticancer properties. Given the diverse beneficial effects of rutin, its efficacy against cisplatin-induced cardiotoxicity has been investigated [11]. It has been reported that the therapeutic effect of cisplatin significantly increases with an increase in dose [12] but at the cost of severe adverse effects [13]. The data regarding the preventive effect of rutin against dose-dependent, cisplatin-induced cardiotoxicity is scarce. Therefore, the focus of our study was to evaluate the protective effect of rutin on cisplatin-induced cardiotoxic effects in isolated perfused rat hearts.

## Materials and methods

Male albino Wistar (250-350 g) rats were randomly divided into five groups (n = 8). This study followed the guidelines of the National Committee for Ethics and Care of Experimental Animals and was approved by the research ethics committee (REC) of King Saud University, Riyadh (SE-19-119). Animals were anesthetized with urethane, and their hearts were removed and mounted on Langendorff’s apparatus. The hearts were perfused with Krebs-Henseleit solution (composition in mM: NaCl, 118.4; KCl, 4.7; MgSO_4_, 1.2; K_2_HPO_4_, 1.2; NaHCO_3_, 25.0; CaCl_2_, 2.5; glucose, 11.5), gassed with 95% O_2_ and 5% CO_2_ at a constant flow of 10 mL/minute, and maintained at 37°C with LE Thermostat (Panlab, Harvard Apparatus, Holliston, MA, USA). A saline-filled latex balloon connected via a polyethylene tube to a pressure transducer (MLA844, ADInstruments, Sydney Australia) was inserted into the left ventricle, and baseline end-diastolic pressure was set at 5-10 mmHg. The pressure transducer was connected to ADInstruments Powerlab 8/30 via a bridge amplifier (ADInstruments, Sydney Australia).

The perfusion pressure was monitored by a second pressure transducer (MLA844, ADInstruments, Sydney Australia) connected to ADInstruments Powerlab 8/30 via a bridge amplifier (ADInstruments, Sydney Australia). Initially, all hearts were perfused for 15 minutes for stabilization. Following stabilization, cardiac functions such as left ventricular pressure (LVP), heart rate (HR), dp/dt (maximum), dp/dt (minimum), perfusion pressure, pressure-time index, contractility index, and duration of diastole were recorded. Results were analyzed using LabChart Pro 7 software. Measurements were carried out one minute before and 60 minutes after perfusion of various cisplatin doses (Ebewe Pharma, Austria). For each dose of cisplatin used in the experiments, a separate isolated heart preparation was employed to avoid the cumulative toxic effects of cisplatin.

In parallel experiments, rutin trihydrate (Sigma-Aldrich GmbH, Germany) was perfused for 10 minutes before administration of 7 or 14 mg/L of cisplatin in the perfusion fluid and throughout the experiment. A third sham group was perfused with Krebs-Henseleit solution for 60 minutes. Responses obtained 60 minutes after perfusion were expressed as percentages in relation to those obtained one minute before perfusion of cisplatin.

Histopathological examination

At the end of the experiment, specimens of the ventricles of control, cisplatin 14 mg/L, and rutin and cisplatin-treated animals were dissected. Ventricle specimens were divided into two portions to analyze histopathological and endogenous antioxidants. The first specimens were fixed in 10% buffered formalin and processed with paraffin wax. For histopathological feature examination, 5 µm sections were stained with hematoxylin and eosin (H&E) for analysis under a light microscope.

Analysis of endogenous antioxidants

The heart specimens were washed with phosphate-buffered saline and stored at -80°C. For the quantification of antioxidant/pro-oxidant variables (glutathione and malondialdehyde), each specimen was weighed, homogenized in relevant specified buffers, centrifuged, and processed following the methodology of sample preparation for each estimate.

Reduced Glutathione Assay

The assay is based on the reaction of reduced glutathione with 5.5-dithiobis-(2-nitrobenzoic acid) (E. Merck Ltd., Mumbai, India), according to the method of Owens and Belcher [14]. The absorbance was measured using a Shimadzu double-beam spectrophotometer (UV200S, Japan) at 412 nm. The concentration of glutathione was calculated using a standard solution of glutathione containing 1 mg of GSH/mL of 3% meta-phosphoric acid. The increase in the extinction at 412 nm was proportional to the concentration of GSH and was expressed as nmol/g wet tissue.

Malondialdehyde Assay

The assay is based on the reaction of malondialdehyde with thiobarbituric acid (E. Merck Ltd., Mumbai, India), according to the method described by Buege and Aust [15]. The concentration of the malondialdehyde-thiobarbituric acid complex was quantified spectrophotometrically at 532 nm and expressed as nmol/g wet tissue.

Statistical analysis

Data are reported as mean ± standard error of the mean (SEM). One-way analysis of variance followed by Tukey’s multiple comparison tests as a post hoc test were used for data analysis. P-values of 0.05 or less were considered statistically significant. Data were analyzed using GraphPad Prism 5 software.

## Results

Effect of cisplatin (1, 7, and 14 mg/L) on the mechanical performance of isolated perfused rat hearts

Table 1 shows the effects of different concentrations of cisplatin (1, 7, and 14 mg/L) on LVP, dp/dt (maximum), dp/dt (minimum), HR, contractility index, and pressure-time index. Eight isolated hearts were perfused with Krebs solution containing different concentrations of cisplatin for 60 minutes. The response obtained at 60 minutes after perfusion was calculated as a percentage of the response obtained at one minute before perfusion of hearts with cisplatin. All concentrations of cisplatin induced a statistically significant (p < 0.01) and dose-dependent reduction in LVP. Cisplatin at 7 and 14 mg/L significantly (p < 0.05) reduced dp/dt (maximum) and dp/dt (minimum). A bell-shaped concentration-response relationship on HR was observed with cisplatin. Cisplatin at 1 mg/L concentration increased HR significantly (p < 0.01), while 7 mg/L slightly reduced HR. All three concentrations of cisplatin significantly (p < 0.01) increased the contractility index, whereas 7 and 14 mg/L predominantly reduced the pressure-time index.

**Table 1:** Effects of cisplatin (1, 7, and 14 mg/L) on the mechanical performance of isolated rat hearts. Parameters are expressed as percentages of values after 60 minutes versus those at one minute before administration of cisplatin. *p < 0.05; **p < 0.001, a significant difference between cisplatin-treated and untreated hearts. LVP: left ventricular pressure; HR: heart rate

Time/parameters	LVP (%)	+dp/dt (%)	-dp/dt (%)	HR (%)	Contractility index (%)	Pressure-time index (%)
One minute before	100	100	100	100	100	100
Untreated, 60 minutes after	94 ± 9	101 ± 7	92 ± 8	101 ± 7	105 ± 3	92 ± 12
Cisplatin 1 mg/L, 60 minutes after	70 ± 6**	97 ± 12	78 ± 7	145 ± 11**	138 ± 5**	74 ± 8
Cisplatin 7 mg/L, 60 minutes after	69 ± 5**	77 ± 3*	72 ± 5*	85 ± 6	140 ± 6**	68 ± 7*
Cisplatin 14 mg/L, 60 minutes after	65 ± 4**	72 ± 5*	69 ± 4*	104 ± 7	151 ± 7**	57 ± 7*

Effects of rutin trihydrate (1 µM) on mechanical performance altered by cisplatin

Table 2 shows the effects of rutin trihydrate (1 µM) on the alterations of the mechanical performance of isolated rat hearts induced by cisplatin (14 mg/L). Rutin trihydrate (1 µM) was perfused 10 minutes before the administration of cisplatin and continued throughout the experiment. Rutin (1 µM) significantly (p < 0.05) reversed the cisplatin-induced (14 mg/L) reduction of LVP and reduction in dp/dt (maximum) and dp/dt (minimum).

**Table 2:** Effects of rutin trihydrate (1 µM) on the mechanical performance altered by cisplatin (14 mg/L) in isolated rat hearts. Rutin trihydrate was administered 10 minutes before administration of cisplatin (14 mg/L) to eight isolated hearts. Parameters are expressed as percentages of values after 60 minutes versus those at one minute before administration of cisplatin. *p < 0.05, **p < 0.01: significantly different from untreated; #p < 0.05, ##p < 0.01 significantly different from cisplatin-treated hearts (14 mg/L), 60 minutes after. LVP: left ventricular pressure; HR: heart rate

Time/parameters	LVP (%)	+dp/dt (%)	-dp/dt (%)	HR (%)	Contractility index	Pressure-time index	Diastolic duration	Perfusion pressure
One minute before	100	100	100	100	100	100	100	100
Untreated, 60 minutes after	94 ± 9	101 ± 7	92 ± 8	101 ± 7	105 ± 3	92 ± 12	116 ± 7	166 ± 16
Cisplatin, 14 mg/L, 60 minutes after	65 ± 4**	72 ± 5*	69 ± 4*	104 ± 7	151 ± 7**	57 ± 7*	99 ± 12	117 ± 7
Cisplatin 14 mg/L + rutin 1 µM, 60 minutes after	84 ± 3^##^	85 ± 6^#^	83 ± 4^#^	86 ± 6^#^	101 ± 7^##^	82 ± 5^##^	139 ± 17^#^	84 ± 2^#^

There was a non-significant increase in HR after perfusion for 60 minutes with cisplatin (14 mg/L). Perfusion of hearts with rutin (1 µM) significantly (p < 0.05) reduced HR. Perfusion of the hearts with cisplatin (14 mg/L) for 60 minutes significantly (p < 0.05) increased contractility index which was reversed by the concurrent administration of rutin (1 µM). Cisplatin-induced reduction in the pressure-time index of isolated perfused hearts was also attenuated (p < 0.01) in hearts concurrently perfused with rutin (1 µM).

Hearts perfused with cisplatin (14 mg/L) for 60 minutes exhibited a decrease in the duration of diastole which was reversed with the concurrent administration of rutin (1 µM) (p < 0.05). Perfusion of hearts with cisplatin (14 mg/L) for 60 minutes caused a non-significant increase in perfusion pressure of the isolated perfused hearts. Rutin (1 µM) significantly (p < 0.01) reduced the perfusion pressure of the isolated perfused hearts.

Histological examination

**Figure 1:**
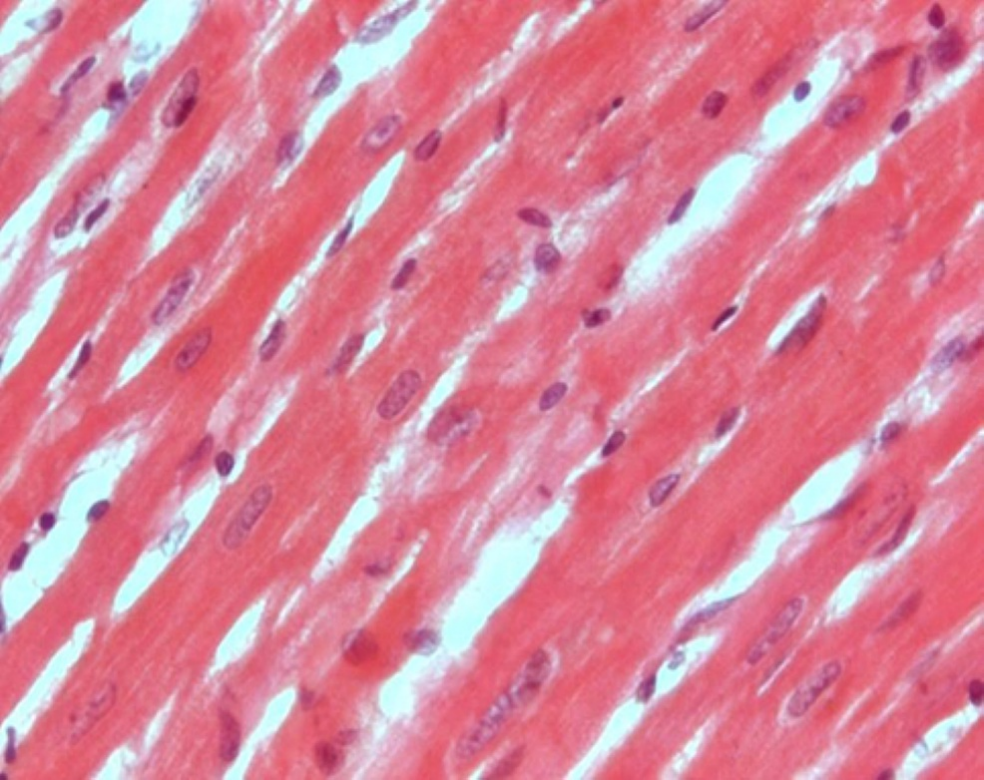
H&E staining of ventricle specimens isolated from untreated (control) rats. The image shows single, oval, and centrally located nuclei of cardiomyocytes with regularly arranged cardiac myofibers. No histopathological lesion was observed in tissue isolated from untreated hearts. H&E: hematoxylin and eosin

**Figure 2:**
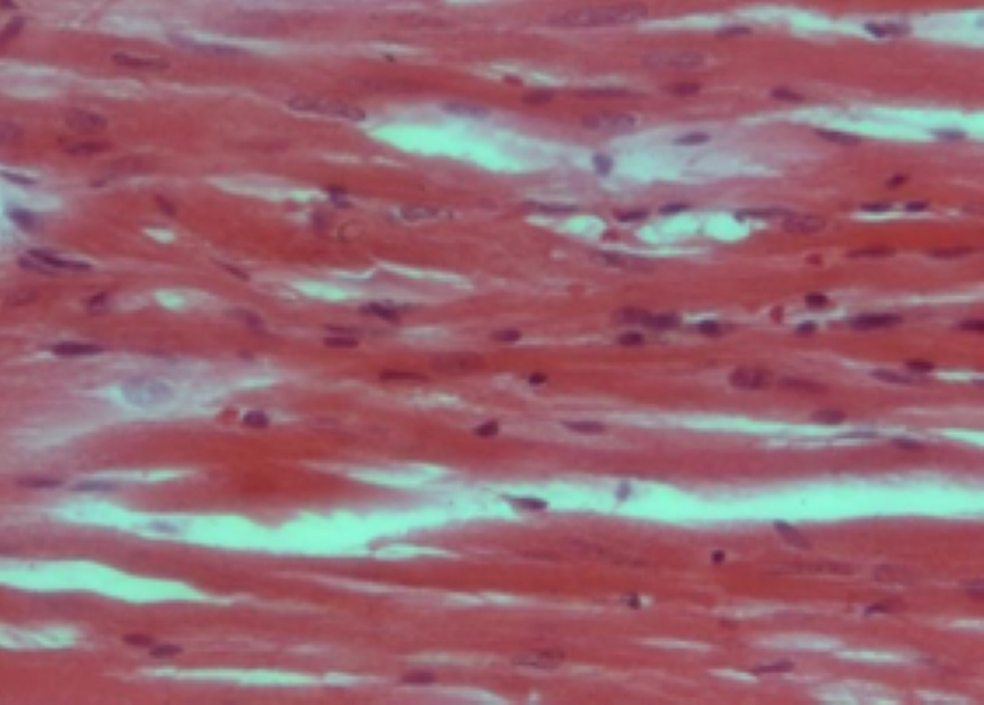
H&E staining of ventricle specimens isolated from cisplatin-treated (7 mg/L) rat hearts. The image shows degenerated muscle cells, necrosis of cardiac myofibers, and dissolution of nuclei in cardiac myofibers. The cardiac myofibers in this group were found to be in a disarrayed pattern compared to the respective untreated hearts. H&E: hematoxylin and eosin

**Figure 3:**
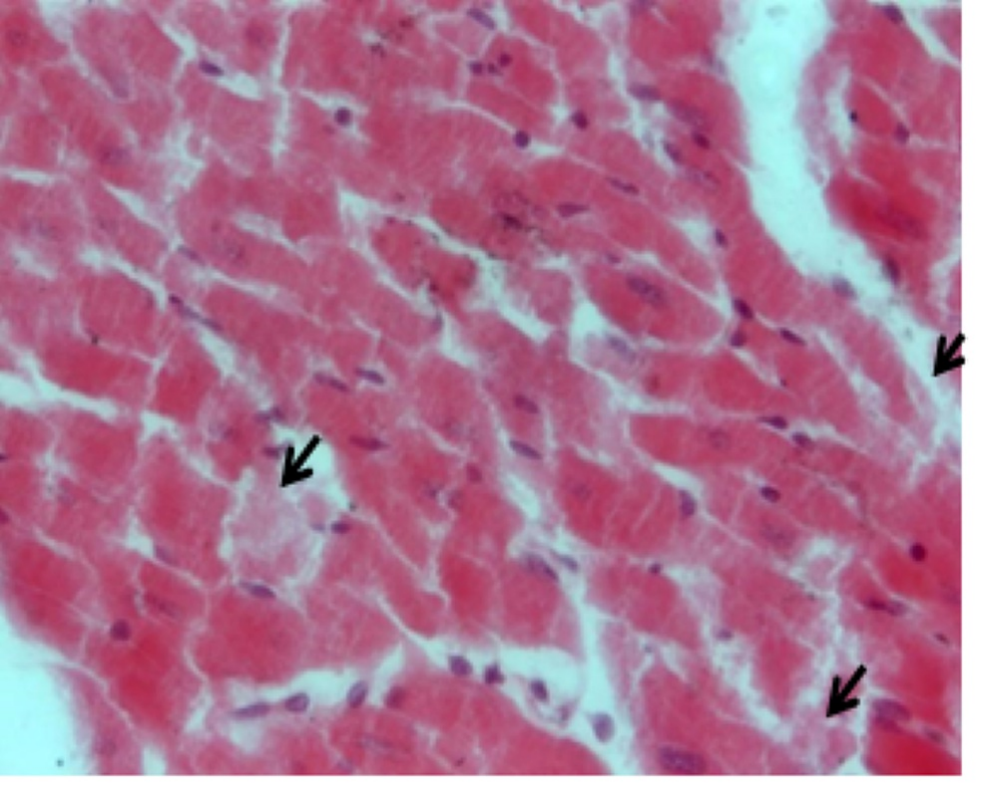
H&E staining of ventricle specimens isolated from cisplatin-treated (14 mg/L) rat hearts. The image shows degenerated muscle cells and interstitial edema (arrows). H&E: hematoxylin and eosin

**Figure 4:**
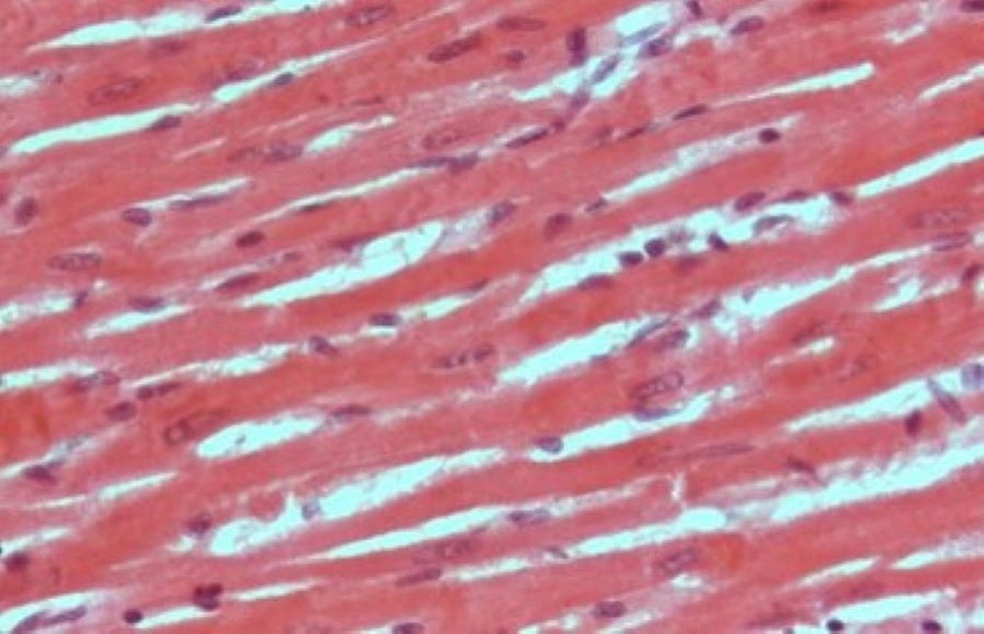
H&E staining of ventricle specimens isolated from rutin 1 µM and cisplatin 14 mg/L (rutin+cisplatin) treated rat hearts. The image shows reduction in cisplatin-induced toxic histopathological effects. Treatment with rutin significantly restored the normal histology. H&E: hematoxylin and eosin

Figure 1 shows the specimens of ventricles from untreated control rat hearts demonstrating a single, oval, and centrally located nuclei of cardiomyocytes with regularly arranged cardiac myofibers. No histopathological lesions were observed in the control group. While specimens from cisplatin-treated (7 and 14 mg/L) rat hearts showed degenerated muscle cells, necrosis of cardiac myofibers, and dissolution of nuclei. The cardiac myofibres in the cisplatin-treated rats displayed a disarrayed pattern compared to untreated control hearts (Figure 2). Ventricle specimens from cisplatin-treated hearts showed degenerated muscle cells with interstitial edema (Figure 3). The addition of rutin (1 µM) to hearts perfused with cisplatin (14 mg/L) reversed the cisplatin-induced toxic changes, and the normal heart histology was significantly restored (Figure 4).

Antioxidant analysis

**Figure 5:**
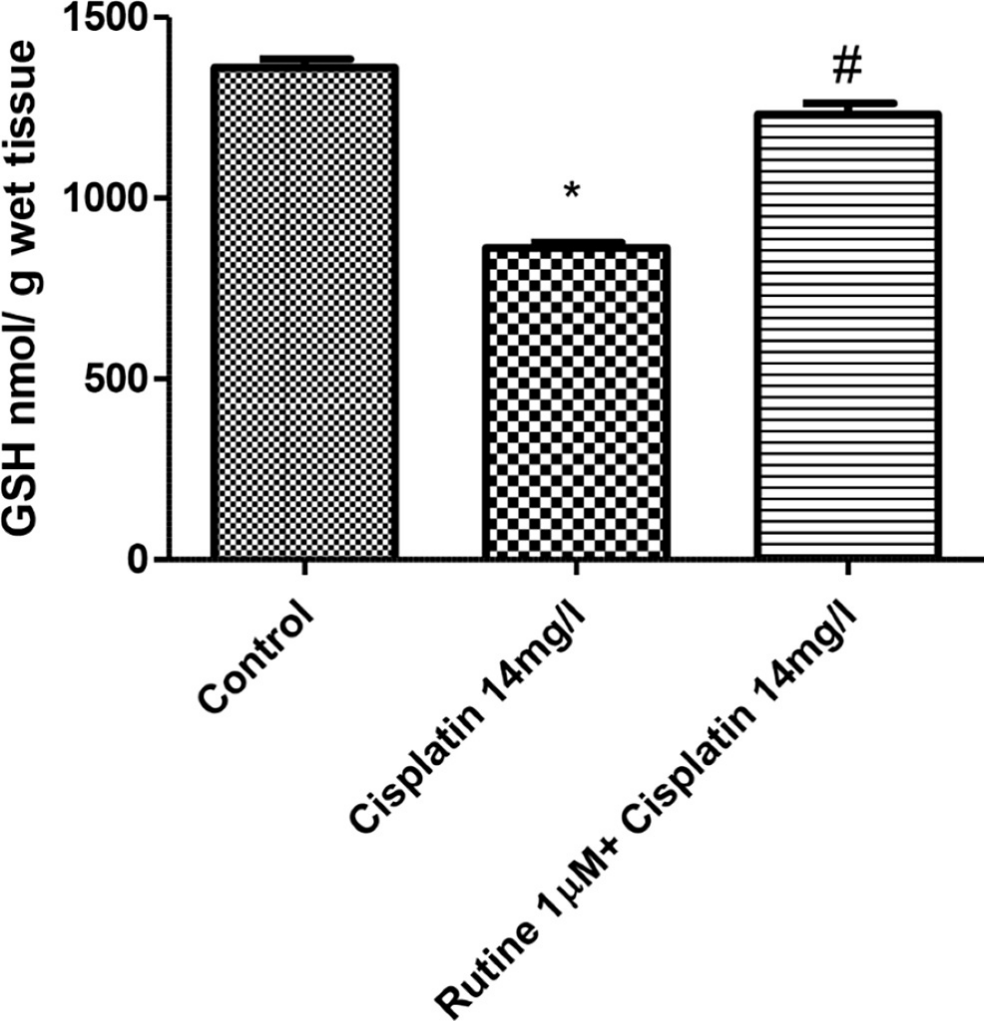
Effect of rutin (1 µM) on reduced GSH concentration in cisplatin-treated (14 mg/L) isolated rat hearts. p < 0.01: significantly different from control; # p < 0.01: significantly different from tissue isolated from cisplatin-treated (14 mg/L) hearts. GSH: glutathione

**Figure 6:**
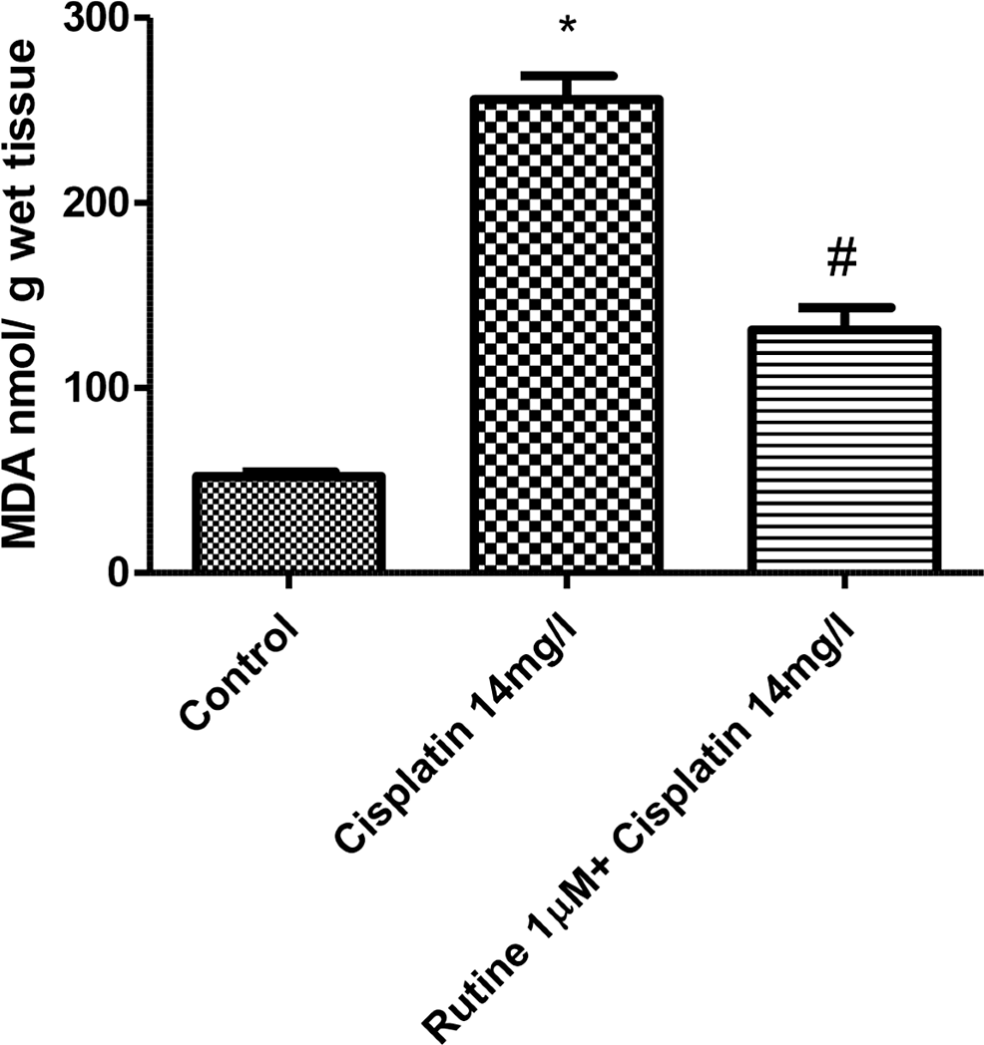
Effect of rutin (1 µM) on increase of MDA concentration induced by cisplatin (14 mg/L) in isolated rat hearts. Effect of rutin (1 µM) on increase of MDA concentration induced by cisplatin (14 mg/L) after perfusion for 60 minutes. Tissues from eight isolated hearts were used to quantify MDA. p < 0.01: significantly different from control, # p < 0.01: significantly different from cisplatin-treated (14 mg/L) hearts. MDA: malondialdehyde

Perfusion of hearts with cisplatin (14 mg/L) significantly (p < 0.01) reduced the glutathione content, while perfusion of hearts with both rutin and cisplatin improved the glutathione content in rat hearts (Figure 5). Perfusion of hearts with cisplatin produced a marked increase in the malondialdehyde content in rat hearts (p<0.01). The addition of rutin to the perfusate containing cisplatin greatly reduced the tissue concentration of malondialdehyde (Figure 6).

## Discussion

Cardiac dysfunction can be assessed by hemodynamic changes in various parameters of cardiac function. In this study, we evaluated the effect of cisplatin alone and with rutin trihydrate on LVP, HR, dp/dt (maximum), dp/dt (minimum), perfusion pressure, pressure-time index, contractility index, and duration of diastole.

Cisplatin caused a significant reduction in LVP, dp/dt (maximum), dp/dt (minimum), and pressure-time index in the isolated rat hearts in a dose-dependent manner after perfusion for 60 minutes. The histopathological findings of heart tissues showed that cisplatin caused degeneration and necrosis of cardiac muscle with the dissolution of nuclei. These findings correlated well with the alterations of hemodynamic and mechanical function induced by cisplatin in the isolated perfused hearts. Additionally, cisplatin increased lipid peroxidation and reduced antioxidant activity in the heart tissue of the animals.

Numerous detrimental effects have been attributed to cisplatin. It has been shown to produce creatine kinase myocardial band and lactate dehydrogenase leakage, a progressive reduction in total carnitine and ATP, and depletion of glutathione content in cardiac tissue [7]. Cisplatin has been shown to cause a dose-dependent reduction in contractile force, heart rate, and coronary flow [16]. Earlier studies have reported oxidative and nitrosative stress, apoptosis, and histopathological alterations with cisplatin in animal experimental models [7,17,18].

Several mechanisms have been proposed to explain the harmful effects of cisplatin on the myocardium. Cisplatin can generate reactive oxygen species, such as superoxide anion and hydroxyl radical [19]. These reactive oxygen species are associated with an increase in lipid peroxidation [20], which results in the leakage of lactate dehydrogenase and creatinine kinase from cardiac myocytes [16]. Moreover, cisplatin-induced reduction in plasma concentrations of various antioxidants [21] can lead to the failure of the antioxidative defense mechanism against free radical-mediated organ damage.

In this study, the aforementioned deleterious effects of cisplatin were evident in the cardiac tissue of the animals. Cisplatin increased lipid peroxidation reflected by the increased tissue concentration of malondialdehyde and reduced tissue antioxidant activity reflected by reduction in reduced glutathione content in cardiac tissue. Glutathione has a direct antioxidant function by reacting with free radicals. Therefore, it is likely that cisplatin-induced toxic effects on the cardiac function and histology of the myocardium could be due to increased lipid peroxidation and reduction of glutathione content in the cardiac tissue of the rats.

Rutin trihydrate has been reported for its beneficial protective effects against cisplatin-induced cardiotoxicity [22]. In this study, the protective role of rutin trihydrate against cisplatin-induced toxic effects on cardiac function such as hemodynamics and histopathological changes was explored. Rutin trihydrate reversed the harmful effects of cisplatin on LVP, dp/dt (maximum), dp/dt (minimum), contractility index, pressure-time index, HR, duration of diastole, and perfusion pressure. Moreover, rutin trihydrate restored the normal histology of the myocardium, inhibited lipid peroxidation, and increased glutathione concentration of the cardiac tissue. Coronary blood flow is impeded during systole. Rutin trihydrate prolonged the duration of diastole and reduced perfusion pressure which could improve the coronary blood flow and subendocardial perfusion.

Cisplatin causes cardiotoxic effects by oxidative stress and by reducing antioxidant enzymes [8]. Rutin trihydrate has been previously reported for its beneficial protective effects against various drug-induced toxicities, including doxorubicin and cisplatin-induced cardiac toxicity [9,10]. The protective effects of rutin trihydrate against cisplatin-induced cardiotoxic effects observed in our study may have occurred due to its marked antioxidant properties. Rutin trihydrate has been shown to enhance superoxide dismutase and 2,2-diphenyl-1-picryl-hydrazyl-hydrate activity [23]. It has also been reported to reduce the cisplatin-induced enhanced lipid peroxide content in rat kidneys [24]. Moreover, rutin trihydrate has anti-inflammatory and anti-apoptotic effects. It inhibits nuclear factor-kappa B and tumor necrosis factor α pathway-mediated inflammation and caspase-3 cell apoptosis [25]. Together, these properties of rutin trihydrate explain its beneficial effects against cisplatin-induced cardiotoxic effects noted in this investigation [11].

## Conclusions

Cisplatin produced a dose-dependent impairment of several parameters of cardiac function such as LVP, contractility index, and pressure-time index. It produced deleterious histopathological alterations in isolated rat hearts. These harmful effects of cisplatin were suppressed by rutin trihydrate, suggesting the potential protective effects of rutin against cisplatin-induced cardiotoxicity. Moreover, rutin trihydrate improved the reduced glutathione content and suppressed the malondialdehyde content in the cardiac tissue of isolated rat hearts, suggesting that the observed beneficial effects of rutin trihydrate in this study could be attributed to its antioxidant properties.

Based on our findings, rutin trihydrate appears to be a potential drug candidate to ameliorate the toxicity of cisplatin and other cardiotoxic drugs. Further research is needed to explore the detailed protective mechanism of rutin trihydrate against cisplatin-induced cardiotoxicity.
